# The Myosin Myocardial Mesh Interpreted as a Biological Analogous of Nematic Chiral Liquid Crystals †

**DOI:** 10.3390/jcdd8120179

**Published:** 2021-12-11

**Authors:** Pierre-Simon Jouk, Yves Usson

**Affiliations:** 1 Equipe Biologie Computationnelle et Mathématique, Univ. Grenoble Alpes, CNRS, VetAgroSup, Grenoble INP, CHU Grenoble Alpes, TIMC Grenoble, La Tronche CEDEX, 38706 Grenoble, France; yves.usson@univ-grenoble-alpes.fr; 2 Service de Génétique, Génomique et Procréation, CHU Grenoble Alpes, CS 10217, CEDEX 9, 38043 Grenoble, France

**Keywords:** cardiomyocytes, myosin, polarised light microscopy, liquid crystals, myoarchitecture

## Abstract

There are still grey areas in the understanding of the myoarchitecture of the ventricular mass. This is despite the progress of investigation methods since the beginning of the 21st century (diffusion tensor magnetic resonance imaging, microcomputed tomography, and polarised light imaging). The objective of this article is to highlight the specificities and the limitations of polarised light imaging (PLI) of the unstained myocardium embedded in methyl methacrylate (MMA). Thus, to better differentiate our method from other PLI modes, we will refer to it by the acronym PLI-MMA. PLI-MMA shows that the myosin mesh of the compact left ventricular wall behaves like a biological analogous of a nematic chiral liquid crystal. Results obtained by PLI-MMA are: the main direction of the myosin molecules contained in an imaged voxel, the crystal liquid director n, and a regional isotropy index RI that is an orientation tensor, the equivalent of the crystal liquid order parameter. The vector n is collinear with the first eigenvector of diffusion tensor imaging (DTI-MRI). The RI has not been confounded with the diffusion tensor of DTI that gives information about the three eigenvectors of the ellipsoid of diffusion. PLI-MMA gives no information about the collagen network. The physics of soft matter has allowed the revisiting of Streeter’s conjecture on the myoarchitecture of the compact left ventricular wall: “geodesics on a nested set of toroidal surfaces”. Once the torus topology is understood, this characterisation of the myoarchitecture is more accurate and parsimonious than former descriptions. Finally, this article aims to be an enthusiastic invitation to a transdisciplinary approach between physicists of liquid crystals, anatomists, and specialists of imaging.

## 1. Introduction

The study of the topological organisation of myocardial cells is a basic requirement for an understanding of the mechanical design of a normal and pathological heart. There are still grey areas in the understanding of the myoarchitecture of the ventricular mass. This is despite the progress of investigation methods (diffusion tensor magnetic resonance imaging, microcomputed tomography, and polarised light imaging) since the beginning of the 21st century [1,2,3].

In 1990, Grenoble-Alpes University had two teams that could contribute to scientific knowledge on this subject. On one hand, the perinatal pathology unit of the genetics department of the CHU Grenoble-Alpes had a collection of normal and pathological hearts legally declared for research on normal and pathological development and in the laboratory; on the other hand, a research team, “Pattern Recognition and Quantitative Microscopy”, with extensive experience in confocal laser microscopy and polarised light imaging (PLI). The association of these two teams within the project “Topography of myocardial cells during perinatal development” made it possible to start this difficult and ambitious project. It is difficult because it is a transdisciplinary project (Physics, Mathematics, and Biology), and it is positioned as a multiscale problem, from cell to organ. Our specific material, i.e., ex vivo perinatal hearts, and the fact that we had developed a suitable polarised light imaging device led us to original methodological choices. These methods were different from those developed by the medical imaging industry for MRI as well as x-ray tomography. They did not lead to contradictory results but required different modes of data representation. This made the comparison between these quite demanding, thus requiring a voluntary effort to assess and integrate our new data. Our motivation of a different approach than that made by the industry was justified by the fact that the previous methods were judicious for the study of the myoarchitecture of the left ventricle but proved difficultly suitable to decipher the myoarchitecture of the right ventricle.

Thus, the objective of this article is to present a comprehensive view of the contribution of PLI to the understanding of the myoarchitecture of the ventricular mass. This is not an extensive review—there is no historical claim. Only main references to the work of the masters of the discipline are given in which you will find the historical perspectives. There are also no mathematical nor physical optics claims, but all the necessary and useful references for the reproduction of the original methods that we have developed over the past 30 years are given.

## 2. Background: Materials and Methods

### 2.1. Ethics Statement

Grenoble University Hospital (GUH) owns a legally declared collection of embedded tissue sections collected after autopsy for perinatal and infant death performed for a diagnostic purpose. Written consent was obtained from the parents or guardians at the time of the request for autopsy authorisation and for research authorisation on normal and abnormal development. The institutional review board of Grenoble-Alpes University Hospital approved the research protocol. Samples dedicated to research purposes were kept anonymous. The study was conducted in accordance with the 1964 Declaration of Helsinki and its later amendments.

### 2.2. Polarised Light Imaging (PLI) and Histological Preparation

The physics of polarised light microscopy for petrographic studies and crystallography was well-established since 1888 by Auguste Michel-Levy, and the first interference colour chart was named after him and is of daily use in all fields of microscopy [4]. During the twentieth century, some research was conducted to adapt PLI for the investigation of biological materials. In 1986, two major syntheses on this subject were published.

The first was a joint study by the Department of Biophysics and the Department of Geology at the University of Western Ontario [5]. It was an adaptation of Michel-Levy’s methods for the study of the muscular tunic of human cerebral arteries and the point-by-point manual measurement of the alignment of the mechanical axis of smooth muscles.

The second was a masterful study by Shinya Inoue from the Woods Hole marine biological laboratory [6]. Shinya Inoue demonstrated that charge-coupled device cameras (CCD cameras) could extract all information from the PLI and used to automate the measurement of the orientation of birefringent biological structures of a microscopic tissue preparation. However, at this stage, a problem remained for the study on the myoarchitecture of the perinatal myocardium.

**Figure 1:**
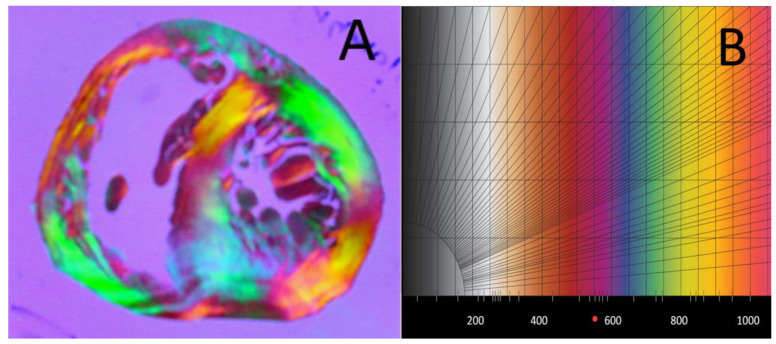
The myocardium embedded in the MMA behaves like a mesh of positive uniaxial crystals of myosin. (**A**) View of a 500 µm thick slice of a neonatal heart embedded in MMA, imaged between crossed polars with the addition of a full wave plate (path difference 540 nm). (**B**) Michel Levy colour chart from 0 to 1100 nm path difference. The comparison of the colours between A and B makes the visual assessment of the phase difference of the rays passing through the crystal possible.

The properties of birefringence of fresh or fixed non-embedded myocardium are complex and heterogeneous. Myocardial birefringence is essentially the sum of two different types of birefringences—the crystalline birefringence of myosin and the form birefringence of the different collagens [7]. The crystalline birefringence is intrinsic to the structural arrangement of myosin, while form birefringence is dependent of the variability of the relationships between the different collagens and with an extracellular matrix. This collagen-form birefringence can be either additive or subtractive to the crystalline birefringence of myosin and makes it impossible to extract accurate information about the 3D-orientation of myosin filaments and, by extension, of myocardial cells. The adopted histological solution was to cancel the form birefringence of collagen by embedding the heart in methylmethacrylate resin (MMA), whose refractive index is close to the refractive index of collagen. Thanks to MMA embedding, myocardial birefringence behaves like a positive uniaxial crystal with a 0° extinction angle (Figure 1). That made it possible to extract the 3D-orientation of myocardial cells with high spatial resolution (100 micrometres) and accuracy [8].

The orientation information is expressed by two angles:

- the azimuth angle (az), that is the angle between the east–west axis of the optical bench stage and the projection of the measured mean orientation of a few thousands of myocardial cells in an elementary point of the image (voxel) on the stage plane (0°–180°);
- and the elevation angle (el), that is the angle corresponding to the obliquity of the voxel principal orientation, with respect to the plane of the section; in other words, it is the way the voxel principal orientation escapes from the section plane (−90° to +90°).

The embedding technique has disadvantages: the processing time to embed a full heart is long (3 months); the polymerization of MMA is exothermic and limits the size of the hearts to be embedded to a maximum of 200 cubic centimetres (size of fetal and small infant hearts); then, the serial sectioning with a rotary diamond saw is time consuming (30 min per section) and therefore onerous.

**Figure 2:**
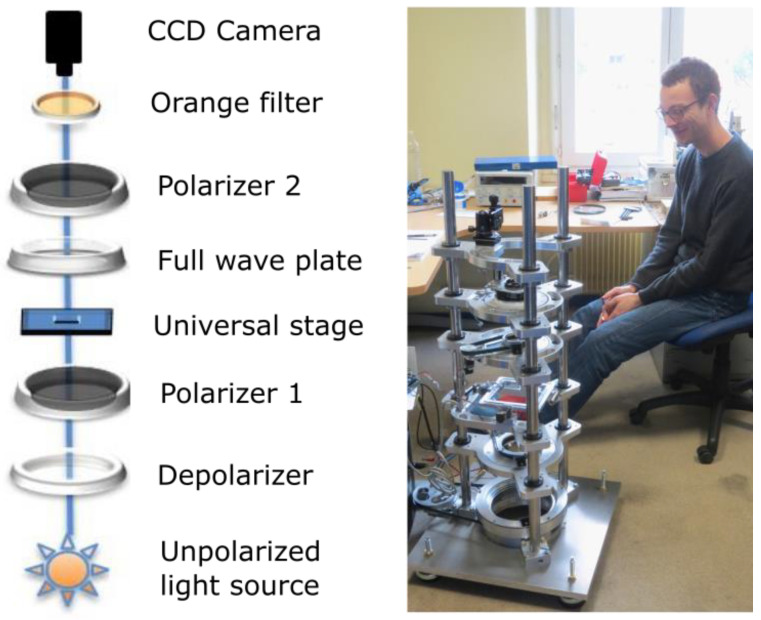
The automated optical bench—the heart slice embedded in MMA is positioned on a universal stage for the acquisition of the forty-nine images necessary to extract the orientation information.

However, it also has advantages because the sections do not require staining, no bleaching or degradation are to be expected, and the sections can be stored for decades. That makes it possible to build collections of serial sections that comply with legal and ethical standards that currently limit prolonged storage of whole organs. These were an advance for our study since we could re-study our section collections each time we improved our polarised light imaging techniques. For example, in 1995, we were only able to measure the orientation angle within a range extending over an eighth of the sphere [9]; by 2000, we extended this range to a quarter of the sphere [10]; and in 2014, to half of the sphere, which is the complete definition of myocardial orientation [11,12]. A corollary effect of these progresses in the angular range was the increasing number of the polarisation image configurations to be acquired—that is, with different combinations of crossed polars and different tilts of the section planes. For example, the calculation of orientation information over an eighth of a sphere requires the combination of information provided by five images each, with a different position of the crossed polars. Extraction in a quarter of a sphere necessitates the combination of information provided by thirteen images each, with a different position of the crossed polars and the full wave plate. Extraction in half of a sphere necessitates the combination of information provided by forty-nine images each, with a different position of the crossed polars of the full wave plate and with different values of the tilt of the universal stage. The transition from determining the quarter-sphere to determining the hemisphere was particularly long since the process of fully automating the acquisition and data interpretation was complex, as well as handling the problem of voxel-to-voxel matching of tilted images. Fortunately, the emergence of affordable consumer automation electronics (e.g., Arduino, Raspberry PI, and step-motor drivers) during the past decade helped us in integrating these functions into a fully automated and efficient optical bench (Figure 2). As a result of all the specificities described above, we will refer now to our PLI technique as PLI-MMA to differentiate them from other modes of polarised microscopy.

### 2.3. Alternative Optical Methods of Studying 3D Myocyte Organisation

Other approaches of polarimetry using Matrix Mueller methods in the case of highly diffusive tissues are a better tool for the characterisation of the microstructure of tissues [13,14,15]. In our case, we used a thin section of a low-diffusive material with a rather homogeneous absorption, inducing a low level of diffusion. Furthermore, we cancelled the depolarising effect of collagens (form birefringency interferes with the myosin crystalin birefringency) by embedding the tissue in MMA, as explained above. However, in future works, the use of the Mueller matrix will probably be very useful to characterise the homogeneity of the orientation measurements within a voxel.

We also tried SHG (second harmonic generation microscopy) 20 years ago, but, unfortunately, the mode of inclusion of our tissues was incompatible with this type of imaging; in fact, MMA did not dissipate the laser power absorbed in 2-photon excitation, and MMA sprays immediately and destroys the sample. For the SHG, it is necessary to work on fresh material, not embedded, and is, unfortunately, incompatible with our collection.

Finally, this project was collaborative and involved many students from the mechanical and physical spectrometry laboratories of the University of Grenoble-Alpes. The impetus was given by the admiration we felt for Auguste Michel-Levy, who spent a good part of his professional life in the Alps, in Chamonix in particular. Eventually, the total amount of the manufacturing and automation project was around 200,000 euros, including thesis and master’s grants.

### 2.4. Mapping

The problem we faced was how to account for and represent angular orientation information as understandable and unbiased maps. Strictly speaking, all 3D coordinate systems are able to define, without ambiguity, the position of a given voxel in space. In the spherical coordinate system, once the centre of the sphere is defined, the coordinates of voxels within the ventricular mass are expressed with a single measure of distance and two measures of angle (r and Thêta, Phi). In the cylindrical coordinate system, once the longitudinal axis of revolution is defined, the coordinates are expressed with two distances and one angle (z, r and Phi). In the Cartesian system, coordinates are defined by three distances (x, y, and z). Thus, the choice of a coordinate system could be considered neutral. Unfortunately, the choice of a coordinate system often also becomes the choice of a model. This confusion is at the origin of an insidious artefact. For example, the medical imaging industries (MRI, X-ray tomography) have frequently chosen a cylindrical coordinate system and model. This is consistent with the objective they pursued: the simplicity of visual reading of maps for the left ventricle, which presents a coarse symmetry of revolution. However, they additionally decided to take advantage of this cylindrical coordinate system to transform the measured values of the azimuth and the elevation angle (as defined in para 1.2) into values calculated with respect to the plane tangent to the cylinder: the imbrication angle and the helical angle. These calculated values are interesting but model-dependent. They are biased because they depend on the choice of the longitudinal axis of symmetry of the left ventricle that is partly arbitrary (operator-defined). They lead to a cosmetic smoothing of the angular values because they do not take into account the roughness of the contours of the real ventricular walls and the large deviations from the symmetry of revolution. The ellipsoidal coordinate system largely overcomes these drawbacks but is still partially dependent on the model [16].

Therefore, we decided to keep the Cartesian coordinate system, and thus, each voxel of the ventricular mass was characterised by 3 localization values, x, y, and z, and 2 mean orientation values, az (range 0° to +180°) and el (range −90° to +90°).

**Figure 3:**
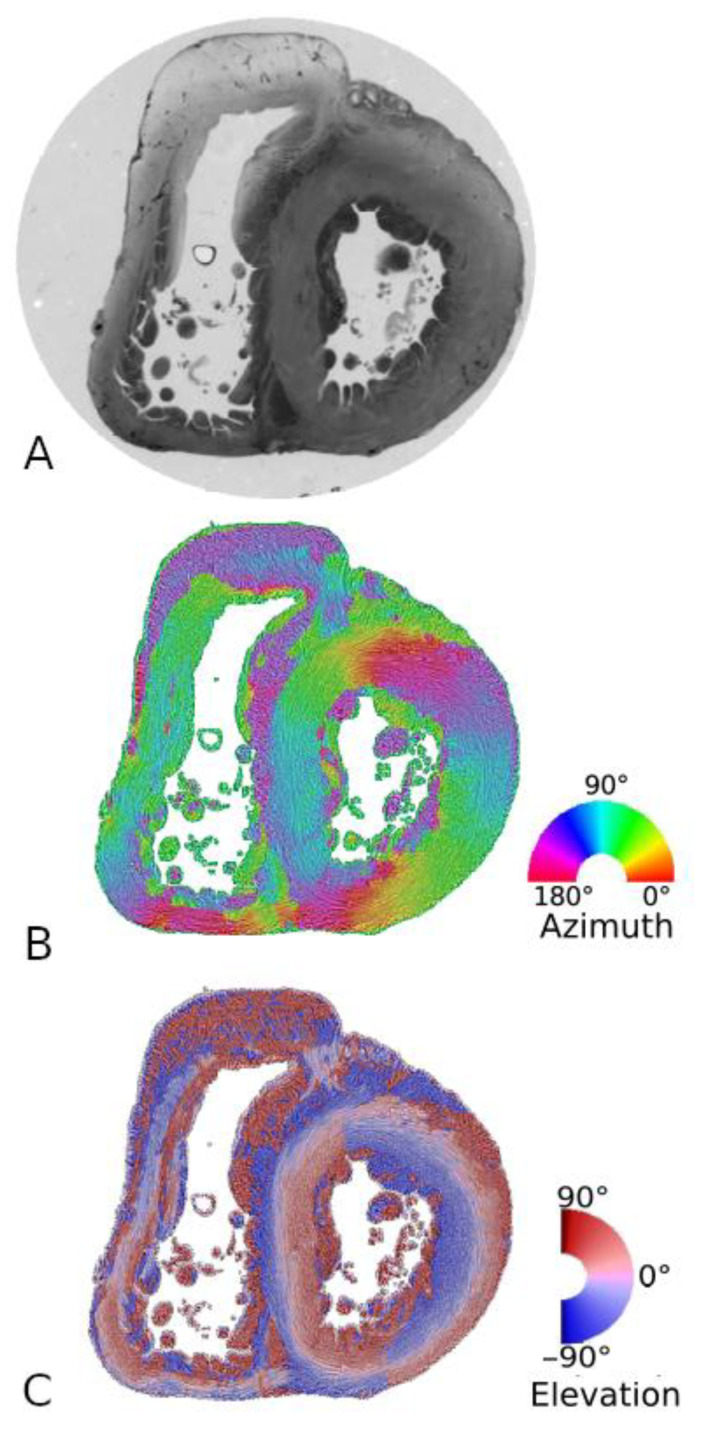
Example of PLI-MMA orientation maps. (**A**): Short-axis section of a neonatal heart, as seen in simple transmitted light after embedding in MMA resin. (**B**): Same section, colour-coded azimuth map with superimposition of LIC texture. (**C**): Colour-coded elevation map with superimposition of LIC texture. The checkered appearance (colour discontinuities at the superior and inferior part of the ventricular wall) visible on the elevation maps are due to the sudden change from 90° to −90°, when the azimuth angle ranges from 0° to 180°.

This has the advantage of an unbiased representation, but it suffers from some classical difficulties common to all representation of circular data: the meridian and anti-meridian discontinuities. These representation difficulties are minimized by superimposing a streamlined representation based on a classical Line Integral Convolution (LIC) algorithm [17]. It creates a textured image built from limited regional tractographies (five voxels span). Such a streamlined representation avoids the problem of the loss of bijectivity for tractographies in large regions (region > 25% ventricular mass) (Figure 3).

### 2.5. Comparison between PLI-MMA and Diffusion Tensor Imaging (DTI) Measurements

**Figure 4:**
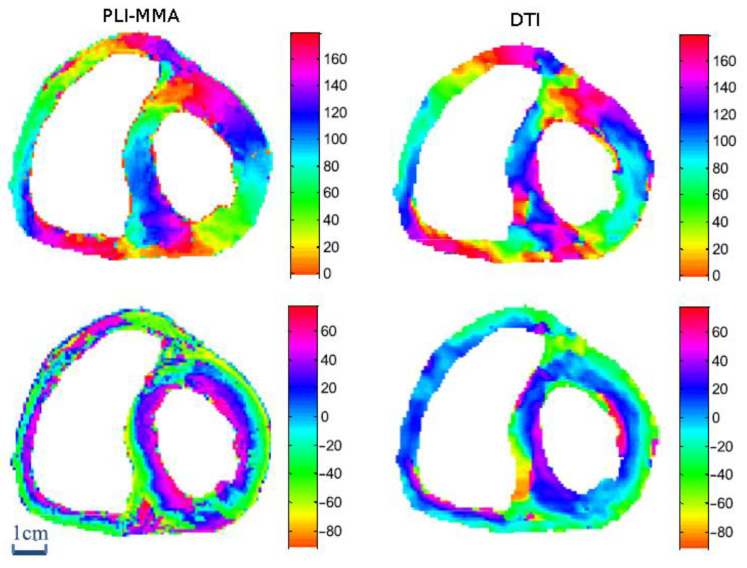
Comparison of the registered DTI and the downsampled PLI maps of an equatorial heart section. Upper row: azimuth maps; Lower row: elevation maps. For the sake of comparison, the representation in cartesian coordinates of PLI elevation maps was transformed in cylindrical coordinates that are the rule for DTI representation.

With PLI-MMA, the orientation of myocardial cells is inferred by the measurements of the principal orientation of the myosin mesh; with DTI, this orientation is inferred by the primary eigenvector of the diffusion tensor of water molecules. Excellent correlations have been found between these two methods (Figure 4), especially when the spatial resolution of PLI-MMA is reduced to the significantly lower values of DTI [18].

However, DTI also provides the values of the secondary and tertiary eigenvector of the diffusion tensor for each voxel. That makes it possible to compute a diffusion tensor index—the fractional anisotropy index (FA). PLI-MMA cannot extract the secondary and tertiary eigenvectors, thus it will not bring new arguments about the myocardial aggregates knowledge. However, the characterisation of the voxel-to-voxel variations in orientation within the entire ventricular mass is provided by computing a Regional Isotropy index (RI). The latter varies from 0 for complete anisotropy, neighbourhood voxels have the same orientation as the centre voxel, to 1 for complete isotropy; that is, all possible orientations are observed in the neighbourhood voxels [19]. This PLI-MMA orientation tensor, RI, must not be confused with the DTI diffusion tensor nor the structure tensor (x-ray CT), although they all account for scattering information.

## 3. The Insights of 1979 Streeter’s Conjecture: “Fibres Run like Geodesics on a Nested Set of Toroidal Bodies of Revolution”

### 3.1. Validation by PLI-MMA of 1979 Streeter’s Conjecture

In many ways, 1979 Streeter’s article is a very difficult article to grasp [20]. First, it is difficult to access; few libraries have this specific volume of the handbook of physiology. Secondly, it needs a graduate mathematical background to be fully understood. In addition, unfortunately, Streeter dealt with two distinct problems: the anatomy of the entire ventricular mass and the myoarchitecture of the left ventricular wall. The first objective was not original; it obscured the second objective that is demanding in terms of geometry mastering. However, in this handbook article, Streeter brought more geometrical concepts and organisation insight than in all his previously published peer-reviewed papers. In 1969, Streeter et al. [21] described the arrangement of cardiomyocytes in the compact left ventricular wall (LVW) as a crossing double-helical pattern: myocytes in the subepicardium have a negative or left-handed helix angle and those in the subendocardium a positive or right-handed helix angle, whereas those in the midmyocardium are circumferential. This description was not anatomically satisfactory. It did not deal, in particular, with the relations between the different layers of myocytes as convincingly as the Triebwek proposed by Krehl did [22]. Krehl’s proposition inferred the existence of an imbrication angle, now called intrusion angle, that was measured by Streeter with an ingenious histological rotation-section technique. This technique aims at the same purposes as those pursued by Lunkenheimer et al. [23]. Finally, Streeter gave a first validation of Krehl’s Triebwek in his 1979 publication. Richard Peskin, a mathematician specializing in 3D differential geometry, validated the main part of Streeter’s mathematical work in 1989 [24]. However, the sampled areas in the left ventricular wall by Streeter in 1979 were scarce, as were their gross relative localisations. Eventually, we validated Streeter’s conjecture by an extensive study of the whole left ventricle, thanks to PLI-MMA [25]. The mathematical foundation of this article [18] was based on the PhD dissertation of Ayman Mourad [26], who extended and refined the previous work of Charles Peskin [27].

**Figure 5:**
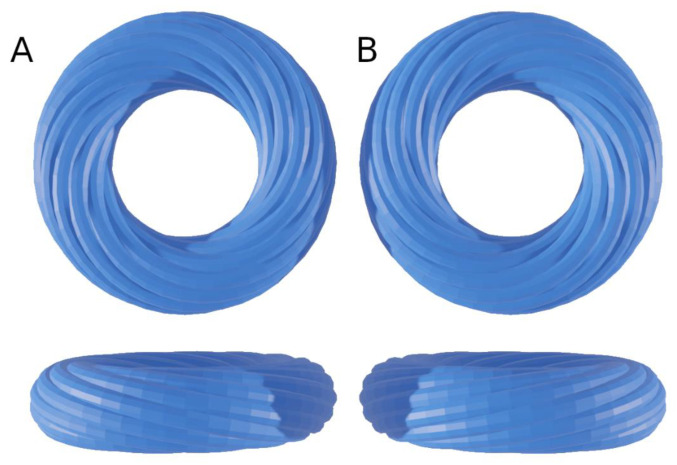
Graphical examples of tori covered with geodesics. Geodesics are pictured as close spaghetti wrapped around the torus; they wrap around the small circumference each time they wrap around the large circumference. A regular curve is right-handed when the current point moves away from the centre; the radius vector rotates counter-clockwise [28]. Column (**A**) Right-handed torus covered by geodesics. Column (**B**) Left-handed torus covered by geodesics. Top row: superior or inferior view (both are identical). Bottom row: lateral view.

**Figure 6:**
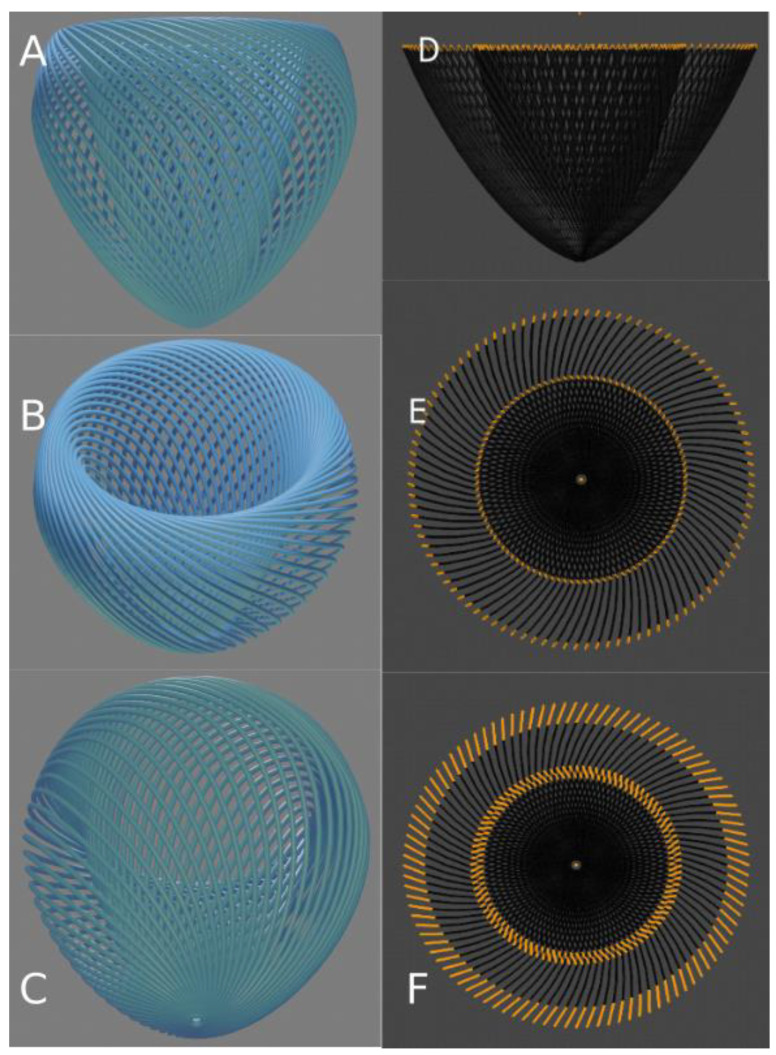
3D model of a single torus, shaped to the compact myocardium of the left ventricle. (**A**) Side view of the torus regularly covered with fifty geodesics (nested tori inside are not displayed). On this torus, the external part represents the sub-endocardial region and the internal part, seen by transparency, represents the interface between the compact myocardium and the trabeculations. The torus aperture is wide in the basal part of the ventricle and becomes almost closed at the apex. (**B**) Three-quarter view from above of the same sketch to visualise the continuity of the geodesics from the inner and the outer part of the torus at the basal level. At this basal level, the mitral and aortic valves would reside. (**C**) Three-quarter view from below to visualise the continuity of the geodesics at the apical level. One can observe the geodesics invaginating at the apex from the outer surface of the torus to its inner surface. (**D**) Side view of an apical section. A hundred geodesics cut are coloured in orange. (**E**) Top view of the same section as in (**D**) with minimal thickness. (**F**) Same as in (**E**) with increased thickness. The segments, viewed from the top, constitute two coronas. The external corona is with a clockwise orientation and the internal one with an anticlockwise orientation. This has been described as a double spiral organisation.

Let us summarize the 1979 Streeter’s article as follows: the definition “geodesics on nested torus” remains very obscure to the anatomist or the cardiologist and deserves to be clarified a bit. A geodesic is the shortest path between two points of a surface. A torus is the surface of a donut (Figure 5) or better, an air chamber that can be stretched to cover and stick to the external contours of the LV and to the trabeculata-compacta interface (Figure 6).

**Figure 7:**
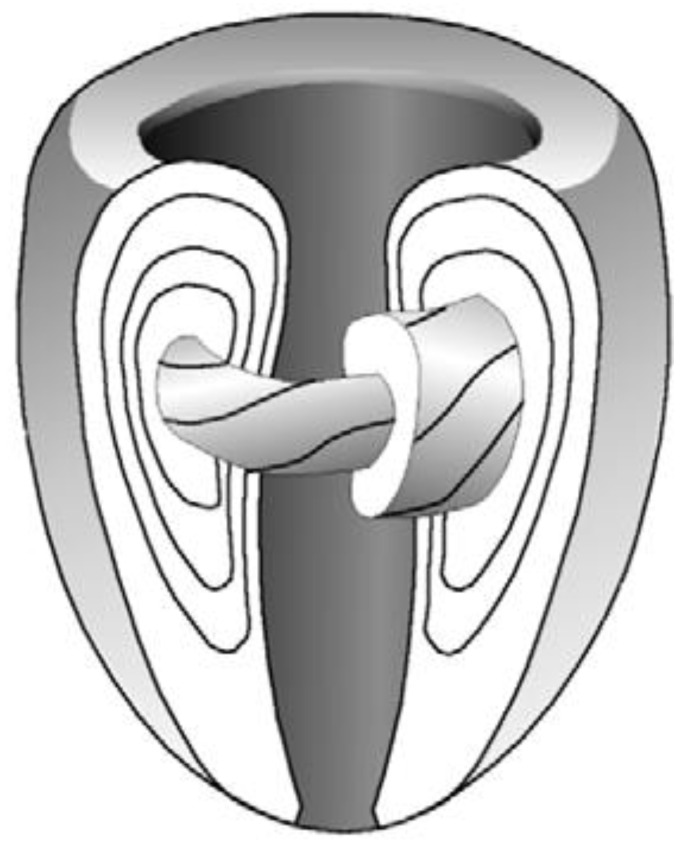
Sketch of the Streeter model “fibres run like geodesics on a nested set of toroidal bodies of revolution” (after 16).

In topology, deformations do not modify the topological characteristics of the considered surface as long as they do not require to be cut using a virtual chisel. Once the greater torus is designed, it is possible to nest smaller tori inside (Figure 7).

One of the consequences of Streeter’s model that was often left aside is that the toroidal model weakens the “double spiral” model of cardiomyocytes in the LVW, since it is only a direct sectioning artefact of the “geodesics on nested torus” model. This can be understood with a simpler model based on a single torus (Figure 5) and becomes more evident in a model built on nested tori. The “double spiral” appearance emerges from the sectioned view of the toroidal model.

### 3.2. The Integrative Value of Nested Tori Streeter’s Model

**Figure 8:**
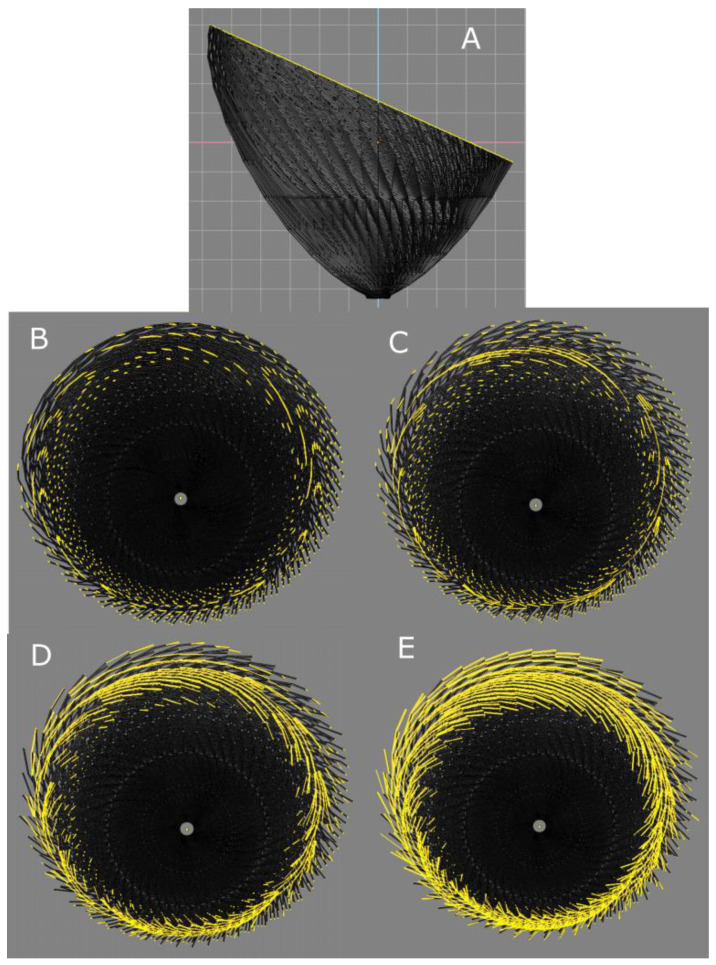
Examples of paralines and emergence of ortholines. 3D model—ten nested tori, each covered by one hundred geodesics. Theoretically, a geodesic is a curve and has no thickness, but, for the sake of representation, we gave it an arbitrary thickness or diameter as if it were spaghetti. (**A**) Side view of an oblique section of a set of ten nested tori, each covered by one hundred geodesics (object size 100 mm). (**B**–**E**) Slices with different thicknesses. The views are perpendicular to the section plane in A. (**B**) Minimal thickness slice: ortholines are resulting from the alignment of a few distinct geodesic segments. They made the measurement of an intrusion angle with the epicardial tangent plane possible. (**C**) Slice with same thickness as the diameter of the geodesic. This extends the length of in-plane geodesics, but the pattern becomes messy. (**D**) Slice with a thickness equivalent to three geodesic diameters. Emergence of paralines, i.e., the end-to-end juxtaposition of geodesic fragments running on different tori. (**E**) Slice thickness of five geodesic diameters.

Daniel Streeter spoke very little about the structure of the myocyte aggregates. Since then, there have been many explorations on this subject, but it remains difficult to reconcile all the existing models. This is in part due to the difficulties in translating Streeter’s geometric language into anatomical representations. For educational purposes, we will try to clarify things by using a digital model of the left ventricular mesh as geodesics on nested toroidal bodies. We know that “all models are false but some are useful” [28], and we hope that ours will be. To do this, we built a model of ten nested toroidal surfaces with axial symmetry, each bearing one hundred geodesics. Each time the geodesic travelled one turn of the small circumference of the torus, it also travelled one turn of the large circumference of the torus. The minimal distance between each geodesic was equal to the diameter of the geodesic (Figure 5). With this model, when making virtual slices of the geodesics, it is possible to highlight geodesics in the plane of the section and to measure the intrusion angle relatively to the epicardial surface (Figure 8). While progressively increasing the thickness of the virtual section, what appeared to be a clear geodesic curve took on the appearance of a segment with increasingly long barbules. This is a classical microscopical artefact with two aspects. On one hand, there is an erroneous mental reconstruction, an optical illusion, trying to create a continuity from superimposed independent segments; on the other hand, we must ask what the substratum of this superposition is. This type of pseudo-organisation resulting from the juxtaposition of many small elements is well-known and characterised by botanists. While studying flowers and fruits (such as sunflowers, pineapples, and pine cones), they were able to distinguish several series of contiguous elements: a main one they called orthostich, along the same plant rod, and secondary ones relying near contiguous points on different rods they called parastich. This is becoming an active field of experimental physics and mathematics [29,30]. However, there are too many differences between vegetal and animal networks to paraphrase botanists, and we propose, in this article, to call “ortholine”, a series of contiguous points on a geodesic, and “paraline”, a series of near contiguous points between different geodesics on different tori.

What is the relationship between ortholines, paralines, and the ventricular mesh?

**Figure 9:**
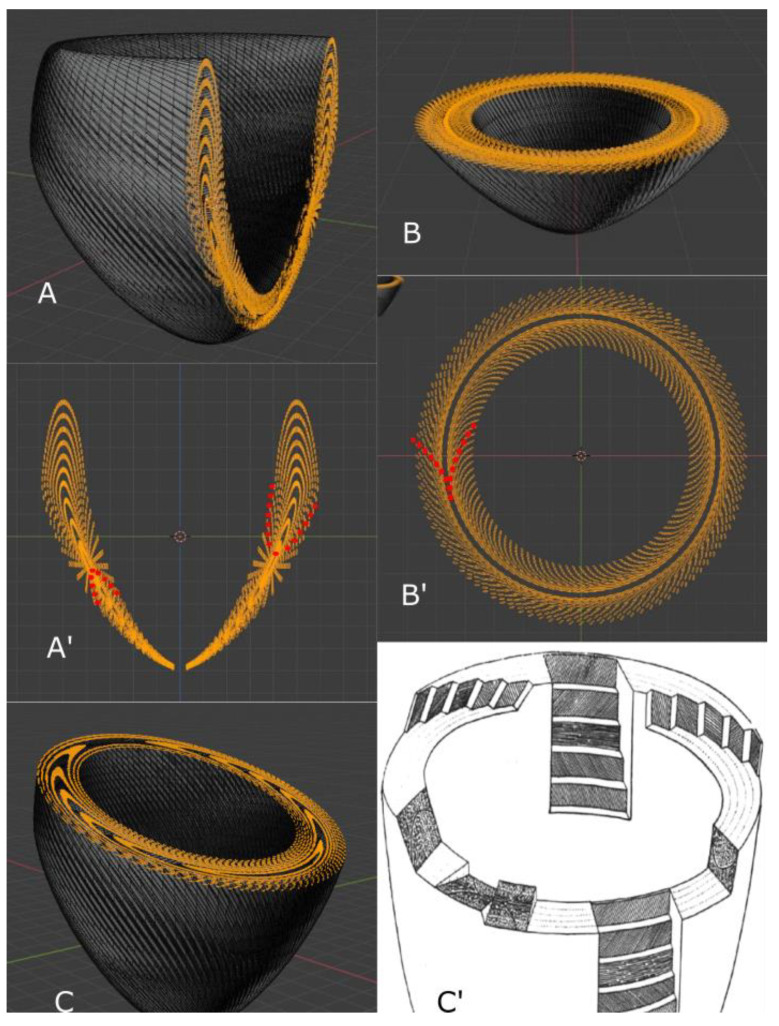
(**A**) Parasagittal section of a set of ten nested tori, each covered by a hundred geodesics. (**A’**) Sagittal slice: Each orange point represents the section of one geodesic. Two ordered series of contiguous orange points draw discontinuous lines: on one are the ortholines that draw the contours of the nested tori and on the other are the paralines that connect the closest neighbouring points between different tori. Two paralines are coloured red to highlight the different orientation of their concavities on either side of the equator. (**B**) Near apical section of the same set of nested tori. (**B’**) Short axis horizontal slice. The ortholines are difficult to identify, with the exception of the two medial wall ortholines that correspond to the inner and outer wall of the deepest nested torus. The paralines describe chevron-shaped patterns, with one coloured in red (**C**) Oblique section: the chevron-shaped patterns vary in their orientation according to their different positions around the revolution axis of this very simplified model of the left ventricle. (**C’**) Reproduction of a 1943 Feneis sketch. The nested tori model explains the observed variations of the different patterns.

The answer is found in the comparison of the different planes of section made in the nested tori model, as sketched by Heinz Feneis in 1943 [31] (Figure 9).

**Figure 10:**
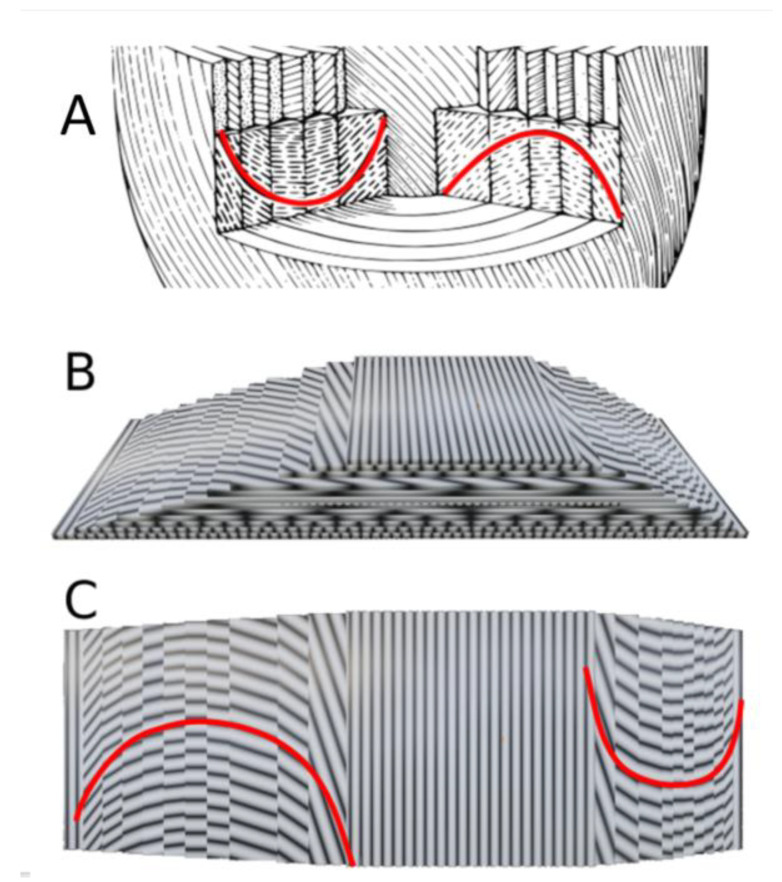
Comparison between a sketch modeled after Feneis and a sketch modeled after Bouligand. Arches highlighted in red in both diagrams illustrate the similarity of sectional artefacts seen in the ventricular myocardium and in the integuments of arthropods. (**A**) Feneis-like Sketch of the ventricular wall. (**B**) Bouligand-like sketch of the integuments of arthropods. The cuticle was cut obliquely on two sides to make a pyramid in order to reveal the plywood organisation and the progressive rotation of chitinous rods. (**C**) Top view of (**B**).

The arch and cusp patterns are constituted by the paralines on each side of the ortholines. These artefactual patterns were well-recognized by Georges Friedel, a physicist working on liquid crystals, as soon as 1922 [32]. It is from these emerging patterns that physicists infer the real structure of liquid crystals. This has been done initially for molecular structure [33], then by biophysicists interested by what they called biological stabilized analogous of liquid crystals [34], and a more complete review can be found in [35]. In Figure 10, we compared the Feneis drawing with a sketch of sections of the arthropod cuticle (after Bouligand). Both sketches show a plywood-like structure characterised by a smooth change of direction between layers through the compact LVW for Feneis and the chitinous fibrous material of the arthropod shells for Bouligand. The visual patterns highlighted in red (cusps and arches) are optical illusions which are characteristic of a chiral nematic semi-liquid crystal, formerly called cholesteric crystal since it was first described in several cholesterol esters. Nematic liquid crystals are composed of rod-shaped structures that maintain a long directional order (*nematos* means thread in Greek) in which the optic axis is parallel to the director and corresponds to what we observed in PLI-MMA. In a nematic chiral, the director rotates steadily throughout the structure, as can be seen in a series of parallel planes.

At this point, we have to quote 1979 Streeter’s conclusions that, “The heart wall was shown to be a 3D continuum made up essentially of the one-dimensional rod element, the cardiac muscle cell. The continuum is characterised by a smooth change of direction across the wall of muscles fibres as in opened-up Japanese fan. The muscle fibre was shown to be an anomalous entity in that it is an abstraction of the near-parallel matrix of muscle cells joined into a network by anastomoses; at any one point in the heart wall, a number of fibre paths can be traced. By the statistical criterion of the principal fibre direction at a given point, this anomaly can be accommodated.”

This fits with the definition of a nematic chiral structure, but the concepts and methods, which would have permitted Daniel Streeter to characterise the structure as a nematic liquid crystal, had not yet spread in the community of biologists. Similarly, we were not aware of this analogy with liquid crystals when we started to study the heart myoarchitecture with PLI-MMA back in 1994. Two favourable events allowed us to make the link between the anatomy and physics of crystal liquids. First, PLI-MMA appeared to be the method of choice; as a direct result, it gives the statistical criterion of the main direction of the fibre. This was a requirement that Streeter mentioned in his conclusion. Second, as noted earlier, is the fact that physicists started to consider the emergence of a liquid crystal-like organisation in biological tissues [28,30,31,36].

### 3.3. Prospective Value of Streeter’s Nested Tori Model

Nowadays, the study of biological analogues of liquid crystals has fostered the rise of this topic, and related articles are now released in close succession [36,37,38].

This new trend is a gold mine for all scientists involved in cardiac development and disease.

Along the way, the concept of chirality was added to the liquid crystal analogy in the frame of biological structures. The chirality of a nematic must not be confused with the well-known chirality at the level of molecules and organs. The new level concerned is that of tissue myoarchitecture. The torus on its own does not possess chirality (Figure 4), but the torus covered with geodesics has one [39]. The chirality of the left ventricular tori covered by geodesics is unique and is right-handed (Figure 5). This was verified in all normal hearts studied with situs solitus. In the case of situs inversus, it might be inverted, but this still has to be confirmed. It is also possible that some discrepancies exist. This is a new field of investigation.

In this article, we have introduced two parameters that characterise liquid crystals: the director and the order parameter. Three others can be transposed in biological tissue: the splay, the twist, and the bend that measure the different types of curvature of the nematic and will be of interest for the study of myocardium biomechanics. They are also of interest to the anatomist because they make it possible to understand why a reverse tractography, starting from the point of arrival of a forward tractography, frequently does not lead to the initial point of departure. This is designated in mathematical terms by the lack of bijectivity of long tractographies.

Despite the fact that we did not study collagens with PLI-MMA, we did not overlook the connective tissue, which was better described by others [2,40,41]. However, our descriptive work does not shed light on the dynamics of interactions between collagens and cardiomyocytes during development and in diseases. The study of experimental animal models will be necessary.

## 4. Conclusions

From a purely technical choice of PLI, we arrived at a characterisation of the compact myocardium, according to the terms of the physics of soft matter and liquid crystals. Initially, the physics of liquid crystals was mainly concerned with mineral materials. The gap between mineralogy and the study of organic matter was difficult to bridge. In this article, we have shown that cardiac anatomy is also considered by this new approach. For a first review in this journal on this new and complex subject, we chose a deliberately simplified didactic approach by limiting ourselves to the description of the compact myocardium. In a future article, we will introduce the notions of singularities that will help more completely describe the ventricular mass.
